# Short report: Weight management of children and adolescents with obesity during the COVID-19 pandemic in Germany

**DOI:** 10.1371/journal.pone.0267601

**Published:** 2022-04-29

**Authors:** Nina Eisenburger, David Friesen, Fabiola Haas, Marlen Klaudius, Lisa Schmidt, Susanne Vandeven, Christine Joisten

**Affiliations:** Department for Physical Activity in Public Health, Institute of Movement and Neurosciences, German Sport University, Cologne, Germany; St John’s University, UNITED KINGDOM

## Abstract

The aim of this analysis was to assess the effectiveness of a juvenile outpatient weight management program during the coronavirus pandemic in Germany, which was implemented digitally during the initial lockdown and thereafter under strict hygiene rules (e.g., adapted exercise sessions). Changes in body mass index standard deviation scores (BMI SDS), physical fitness, media consumption, health-related quality of life (HRQOL), and social self-concept of 28 children and adolescents were compared to data of 30 participants before the pandemic. Adjusted mean changes from baseline to follow-up in BMI SDS (M = −0.07 ± 0.30), relative physical fitness (M = 0.0 ± 0.3 W/kg), media use (M = 0.5 ± 2.6 hours/day), HRQOL (M = −1.6 ± 15.3), and social self-concept (M = −3.8 ± 13.2) during the pandemic were not significantly different from those of the pre-pandemic participants (all p > 0.05). Therefore, the results suggest that an adjusted approach to weight management, which combined digital and adapted in-person components to meet hygiene requirements during the pandemic, was as effective as the pre-pandemic program. It could thus be a potential solution to ensure continuity of care for vulnerable children with obesity during the pandemic and the associated restrictions.

## Introduction

Worldwide, countries, including Germany, have imposed restrictive national health measures and lockdowns to prevent the spread of the coronavirus disease 2019 (COVID-19). Due to school closures, social isolation, and the loss of regular routines, children are particularly affected by the pandemic restrictions [1, 2]. Several studies have shown that these restrictions have led to significant changes in children’s lifestyles which include decreased sports activity participation, increased media consumption, more frequent sedentary behavior, heightened anxiety and fear, and changes in sleep patterns and eating behavior [3–7]. To date, there is limited evidence of the impact of COVID-19 restrictions on children’s weight, but a growing number of researchers predict significant increases in childhood obesity because the observed lifestyle changes are important predictors of weight gain and obesity [1, 8–11].

Given the expected increase in the prevalence of obesity after the pandemic and the fact that obesity is a risk factor for a more severe and complicated course of COVID-19 [12], the need for appropriate weight management strategies for children and adolescents—including lifestyle counseling and psychological support—becomes particularly apparent during periods of social distancing [10, 13, 14]. However, like all other services in Germany, outpatient weight management programs were discontinued, or if at all implemented digitally or modified during the pandemic and lockdown period [15]. While initial findings indicate that digitally adapted weight management during the lockdown was effective in adults [16], there is a lack of knowledge regarding the effectiveness of modified approaches to implementing weight management programs during the COVID-19 pandemic in the context of children and adolescents.

Therefore, the present study analyzed data from two cycles of an outpatient juvenile weight management program during the pandemic (2019–2020 and 2020–2021) in Cologne, Germany. This study aimed to compare the effectiveness of the pandemic period program in terms of BMI SDS reduction, stabilization/improvement of physical fitness, health-related quality of life (HRQOL) and social self-concept, and lifestyle changes (i.e., sedentary media consumption) with pre-pandemic program outcomes.

## Methods

### Data sources and intervention description

The Children’s Health InterventionaL Trial (CHILT III) is an outpatient, multicomponent, family-based program at the German Sport University, Cologne, registered in the German Clinical Trials Register under ID DRKS00026785. Based on the pillars of nutrition, physical activity, medical, and psychosocial support, it is a comprehensive outpatient intervention for children and adolescents with obesity between the ages of 8 and 16 years and their families (the program plan including the study protocol and the CONSORT check-list for randomized trials are provided as S1 File and S1 Checklist). The duration is 11 months, which corresponds to one school year, and it begins each August. The structure and contents of the program sessions, which are carried out weekly with the exception of vacation periods, are presented in Table 1. As illustrated, the children are supervised for a total of about 4 to 5 hours per week, and the parents for 2 to 3 hours per week. Program effectiveness in reducing body mass index (BMI) and improving fitness compared to a control group has been demonstrated in earlier studies [17, 18]. When the pandemic restrictions were initiated in Germany in March 2020, the program of 2019/20 was changed into digital delivery by individual video calls and group sessions until the end of the cycle in July 2020. In August 2020, the new cycle began in-person under strict hygiene regulations. There were no joint cooking or family activities, sports took place either outdoors or, in exceptional cases, digitally (e.g., in very bad weather), and contact sports were omitted. In addition, due to the pandemic-related uncertainties, the participation agreement signed prior to the program with families was less stringent, allowing for easier opt-out of the adapted program during the pandemic if necessary. All medical examinations, counseling sessions, and exercise testing were performed in rigid compliance with hygiene rules. Within these protocols, the program could continue during the second lockdown in Germany (November 2020 to May 2021).

**Table 1:** CHILT III program before and during the pandemic.

CHILT III Pre-pandemic Program			Pandemic Program Adjustments	
			1. Cycle: Aug. 2019-July 2020 *Pandemic Adjustments from* *March 16*^*th*^ *(From August to March 15*^*th*^ *no program changes)*:	2. Cycle: Aug. 2020-July 2021
**Nutrition class by Ecotro-phologist**	**Duration/Frequency**	2 x 45 min. per week Alternating: one week for children, the next only for parents, altering with psychological consultation	1 x 45 min. per week	Same as before the pandemic
	**Implementation**	In-person group session	Group session per videoconference	Under strict hygiene regulations°
	**Content**	Information on healthy food, clarifying questions, group discussions, joint cooking/ grocery shopping	No joint cooking/ grocery shopping	No joint cooking/ grocery shopping
**Exercise/ Physical Activity by Sport Scientists**	**Duration/Frequency**	2 x per week (1x60 min. and 1 x 90 min. = 150 min. in total per week)	2 x 30 min. obligatory, 3 x 30 min. voluntarily per week	Same as before the pandemic
	**Implementation**	In-person group session, family session once per month	Obligatory: Synchronous videoconference Voluntary: Asynchronous video sessions, “challenges”	No family sessions
	**Content**	Group and team sports, coordination games, fitness, trust games, self-efficacy	Obligatory: Group exercise/ fitness, exercise testing under strict hygiene regulations Voluntary: Pedometer, Home work-out	Outdoors or, in exceptional cases, digitally, no contact sports, exercise testing under strict hygiene regulations
**Psycho-social Counseling by Social Pedagogue and/or Psychologist**	**Duration/Frequency**	2 x 45 min. per week Alternating: one week for children, the next only for parents, altering with nutrition	1 x 45 min. per month (group), plus at least 1 x 30 min. per month (individually/family)	Same as before the pandemic
	**Implementation**	In-person group session, individual or family session by arrangement	Regular group session per videoconference, irregular individual or family counseling via video/phone call	Under strict hygiene regulations°
	**Content**	Group dynamics, motivation, self-esteem, individual or family consultation	Same as before the pandemic	Same as before the pandemic
**Office hour/Medical Counseling by Physician**	**Duration/Frequency**	1 x 15 min. per week (individually), plus 3 x 45 min. per program cycle (group session)	1 x 15 min. per week, plus 30 min. individual consultation if needed	Same as before the pandemic
	**Implementation**	Regular in-person individual and family session, irregular group sessions for knowledge transfer	Individual or family video/phone call	Individual sessions under strict hygiene regulations°
	**Content**	Weighing, co-morbidities, metabolic and pathogenetic aspects of obesity	No weighing/ medical examination by physician	Same as before the pandemic
**Medical Examination by Physician/ Sport Scientists**	**Duration/Frequency**	At the beginning and end of the program	Same as before the pandemic	Same as before the pandemic
	**Implementation**	In-person, individually	Endline tests performed under strict hygiene regulations°	Under strict hygiene regulations°
	**Content**	Blood pressure measurement, BIA, calipometry, blood sampling, anthropometric data collection, ergometry, exercise testing/ spiro ergometry	No lactate test	No lactate test

° In accordance with the national health requirements in Germany at this time, such as keeping a distance if possible of 1.5m, using a face mask (FFP2), in the case of exercise testing also using gloves, safety goggles and a protective suit, regular hand washing before and after the session, refraining from taking blood samples; CHILT, Children’s Health InterventionaL Trial (juvenile weight management program analyzed in this study)

The CHILT III participants and their parents were informed that their aggregated data would be anonymized and used for analysis and publication according to the principles expressed in the Declaration of Helsinki [19]. Written consent was provided by the participants’ parents. Ethics approval has been granted by the Sports University of Cologne, for the ethic request with the number 107/2014 which was updated in May 17, 2021.

### Study population and sample size

**Fig 1:**
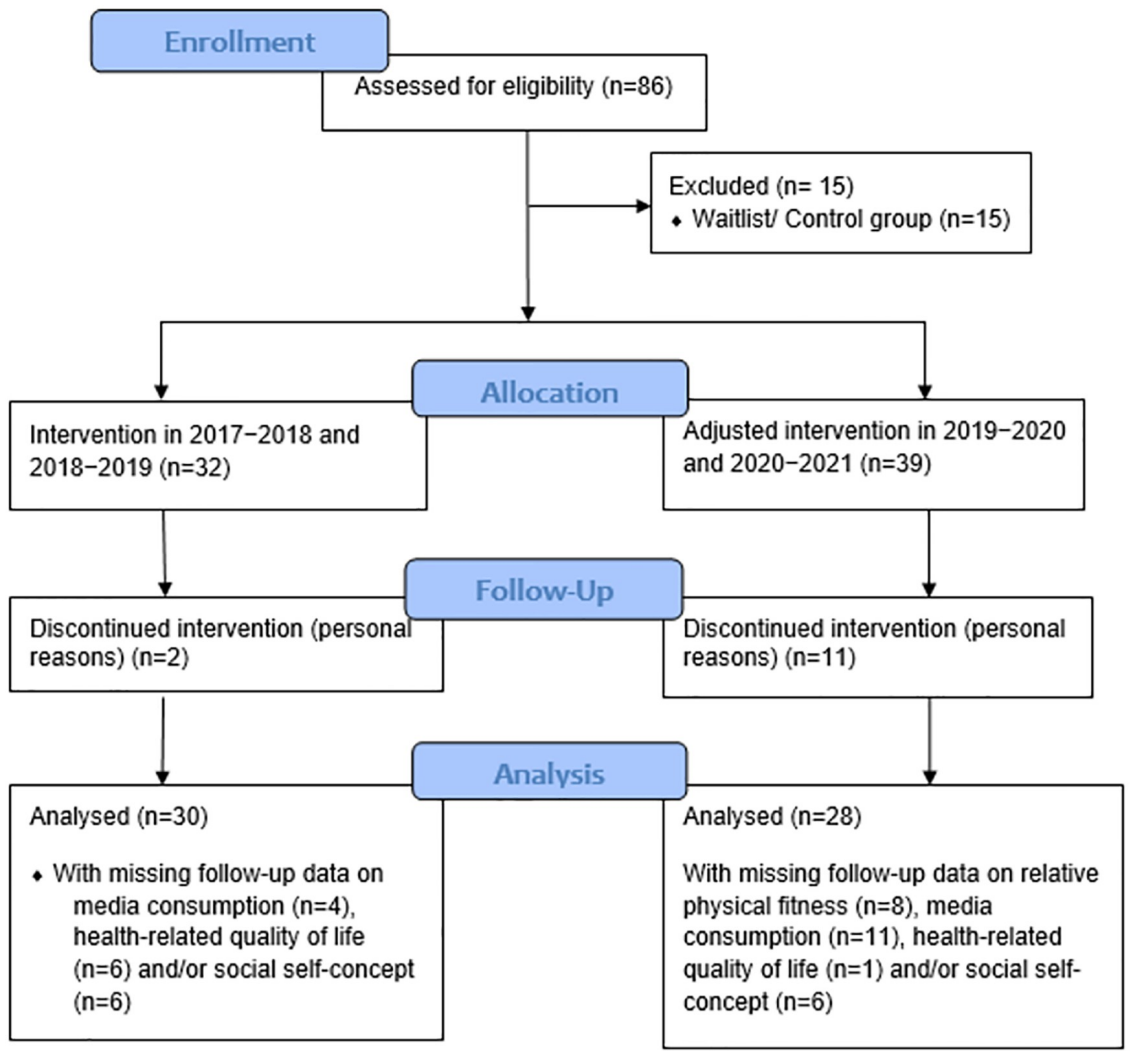
Flow diagram of number of participants in the study.

To compare the children and adolescents who participated in the program during the pandemic with those from earlier years, the sample was clustered into two categories: cluster 1, comprising participants from 2017–2018 and 2018–2019, and cluster 2, consisting of participants from 2019–2020 and 2020–2021 (the groups from 2019/20 and 2020/21 were combined as no significant differences were found between them [see, S1 Table] and in both cases the implementation of the program was partly digitally/modified and partly in-person). In total, the sample consisted of 86 children and adolescents. The minimum requirement per participant to be included in the analysis was participation in the 11-months intervention from beginning to end (as opposed to the waitlist/ control group; Fig 1). Assuming a desired effect size in the medium to large range (f^2^ = 0.06) with a power of 0.61 at an alpha level of 0.05 and an allocation ratio of 1.00, an a priori power analysis for two-tailed independent t-tests performed with G*Power 3.1 showed that at minimum 58 participants were required [20].

### Data assessment

Standard calibrated scales and stadiometers were used to measure and weigh each child with the child barefoot and to assess body mass index (BMI; weight [kg]/ stature^2^ [m^2^]). Sex- and age-specific weight-for-height standard deviation scores (BMI SDS) were calculated according to the German percentile graphs from Kromeyer-Hauschild et al. [21]. At the beginning and end of the program, parents completed standardized questionnaires assessing the demographics and lifestyle patterns of their children. Parent-reported media consumption (i.e., time spent watching television, playing on a game console, using their mobile phone, etc.) was transformed into a continuous variable measured in hours per day. Physical fitness was measured in peak mechanical power (W) using a bicycle ergometer (Ergoline Ergometrics 900). Test results were related to body weight and calculated as W/kg [17]. To assess HRQOL, version B of the weight-specific quality-of-life questionnaire for children and adolescents who are overweight or obese by Warschburger and Fromme was used [22]. A German version of Harter’s Self-Perception Profile for Children by Wünsche and Schneewind was used to assess participant’s social self-concept [23]. After recoding, the highest score for HRQOL and social self-concept was defined as a mean score of 100.

### Statistical analysis

Descriptive statistics are shown as mean values (M) and standard deviation (SD). Independent two-tailed t-tests were conducted to test for significant differences between the clusters in participant characteristics at baseline (t1) using a between-subject design. Program effectiveness was examined by first determining mean changes from baseline to follow-up (t2) in BMI SDS, physical fitness (W/kg), media consumption (hours/day), HRQOL, and social self-concept for both clusters, represented as Δ. Following a between-subject design, mean differences in Δ between the clusters were then compared using analysis of covariance with gender, age, and the respective baseline t1 value as covariates. Homogeneity of regression slopes was not violated with regard to the dependent variables, as the interaction terms between the fixed factor (cluster) and each individual covariate were not statistically significant (p > 0.05). Normality was assumed, upon visual inspection as of the quantile-quantile-plots. The assumption of homogeneity of variances was found to be satisfied, as assessed by Levene’s test (all p > 0.05). Based on visual analysis using boxplots, no extreme values could be identified. Significance was set at p < 0.05. All statistical analyses were performed using IBM SPSS Statistics Version 27.0.

## Results

The final data set consisted of 58 children (51.7% females, age M = 12.5 ± 2.0 years) receiving the intervention from 2017 to 2021. While only two (6%) participants dropped out in the pre-pandemic cohorts, 11 (43%) decided to discontinue the program in the pandemic years without giving detailed explanation. Table 2 presents the baseline descriptive characteristics and differences between the clustered participants during and before the pandemic. The mean BMI SDS of the participants during the pandemic was 2.41 ± 0.49, compared to 2.50 ± 0.45 in the pre-pandemic cohorts. There were no significant differences between the clusters in age, height, weight, BMI, BMI SDS, relative physical fitness, media consumption, HRQOL, and social self-concept at baseline (all p > 0.05).

**Table 2:** Baseline differences between the program participants before and during the pandemic.

Variable	Statistics	Year of participation		p-value
		Cluster 1:	Cluster 2:	
		2017–18 & 2018–19	2019–20 & 2020–21	
t1 Age (years)	n	30	28	0.888
	Mean	12.6	12.5	
	SD	1.9	2.1	
t1 Height (m)	n	30	28	0.831
	Mean	1.59	1.57	
	SD	0.12	0.14	
t1 Weight (kg)	n	30	28	0.665
	Mean	79.8	77.2	
	SD	21.3	24.6	
t1 BMI (kg/m^2^)	n	30	28	0.301
	Mean	31.3	29.8	
	SD	4.9	5.9	
t1 BMI SDS	n	30	28	0.144
	Mean	2.59	2.41	
	SD	0.45	0.49	
t1 Relative Physical Fitness (W/kg)	n	30	28	0.097
	Mean	1.8	1.8	
	SD	0.4	0.4	
t1 Media Consumption (hours/day)	n	29	26	0.773
	Mean	5.9	5.6	
	SD	4.1	3.4	
t1 HRQOL	n	28	25	0.527
	Mean	81.4	79.0	
	SD	14.1	12.7	
t1 Social Self-concept	n	28	24	0.778
	Mean	79.8	81.0	
	SD	13.1	15.6	

t1, baseline data; HRQOL, health-related quality of life; SD, standard deviation; HRQOL and social self-concept are based on scores ranging from 0 (lowest) to 100 (highest); significance values are a result of an independent two-tailed t-test.

Table 3 presents the mean changes in participant characteristics from baseline to follow-up during and before the pandemic. Descriptive analysis of the reported means adjusted for gender, age, and baseline value demonstrated that BMI SDS (mean change = −0.07 ± 0.30), HRQOL (mean change = −1.6 ± 15.3), and social self-concept (mean change = −3.8 ± 13.2) decreased during the 11-month program in the pandemic cluster. Relative physical fitness remained seemingly unchanged (mean change = 0.0 ± 0.3 W/kg) and media consumption per day increased slightly (mean change = 0.5 ± 2.6 hours/day) from baseline to follow-up among 2019–2020 and 2020–2021 participants. All of these adjusted mean changes in the pandemic cluster were not significantly different from those of the pre-pandemic cluster (all p > 0.05).

**Table 3:** Analysis of covariance (ANCOVA) comparing changes in participant characteristics from baseline to follow-up during and before the pandemic.

Variable	Statistics	Year of participation		n_p_^2^	p-value
		Cluster 1:	Cluster 2:		
		2017–18 & 2018–19	2019–20 & 2020–21		
Δ BMI SDS	n	30	28	0.023	0.265
	Mean	0.01	-0.07		
	SD	0.21	0.30		
Δ Relative Physical Fitness (W/kg)	n	30	20	0.015	0.795
	Mean	0.1	0.0		
	SD	0.3	0.3		
Δ Media Consumption (hours/day)	n	25	15	0.017	0.436
	Mean	-0.5	0.5		
	SD	4.3	2.6		
Δ HRQOL	n	22	19	0.019	0.404
	Mean	-2.3	-1.6		
	SD	12.3	15.3		
Δ Social Self-Concept	n	22	13	0.024	0.379
	Mean	1.7	-3.8		
	SD	14.7	13.2		

Reported means are adjusted for age, gender, and baseline value; Δ, difference in data after 11-month intervention (t2) from baseline data (t1); HRQOL, health-related quality of life; SD, standard deviation; p-value represents the significance of *year of participation* as an independent variable in ANCOVA; n_p_^2^, partial eta squared, used as a measure of effect size of the independent variable; HRQOL and social self-concept are based on scores ranging from 0 (lowest) to 100 (highest).

## Discussion

The individual and population-based restrictions needed to mitigate the spread of COVID-19 are undoubtedly impacting children’s lifestyles in unprecedented ways [3, 6, 8, 11, 12]. Woo Baidal et al. reported that children and adolescents with obesity are particularly vulnerable to the negative health consequences of the pandemic restrictions because they already face barriers to healthy lifestyles [10]; social isolation might thus exacerbate existing health inequities [9]. Unfortunately, however, weight management programs for children and adolescents with obesity were also affected by the pandemic-related restrictions [13, 14, 16, 24].

As a consequence, the present study highlights the relevance of innovative approaches to juvenile weight management programs to provide uninterrupted support and to avert the risk of worsening health disparities following the COVID-19 pandemic, even in times of social distancing. According to our results, there was no significant difference in the effectiveness of a juvenile weight management program before and during the pandemic, although important face-to-face components such as group activities (e.g., family sports activities, cooking together) had to be omitted or adapted. Therefore, our findings suggest that, particularly in terms of BMI SDS reduction and stabilization of fitness levels, implementation of the program under strict hygiene restrictions can be considered successful. A combined approach to juvenile weight management including digital and modified in-person components could thus represent an effective way to ensure program continuity in times of pandemic. Even after the COVID-19 pandemic, parts of this approach could be maintained in weight management strategies to increase accessibility and flexibility e.g. through digital counselling [14, 15].

Nonetheless, the implications from our findings regarding a deterioration, albeit marginal, in HRQOL and social self-concept paired with slightly increased media use during the pandemic, which are consistent with previous studies [7, 25], should also not be disregarded. As an increase in screen time, i.e., television viewing for entertainment and social networking, during the pandemic lockdown appears to have negative effects on well-being [26], the use of digital media in medical settings should specifically target social interaction, exercise, and telemedicine care [27]. In addition, weight management programs should consider that while digital content allows for more flexibility, children need daily structure and consistent routines to regulate weight-related behaviors [2]. Therefore, maintaining joint sports activities in the program wherever possible while adhering to hygiene regulations (e.g., outdoor exercise and individual sports) may be essential to promote mental and physical well-being [28].

The results must be interpreted with caution due to high variability in our sample, a small sample size, missing data, and selection bias due to active enrollment in the obesity treatment. The extent to which the program adjustments (including the less stringent participation agreement), strict national hygiene regulations and resulting avoidance of possible contacts led to more dropouts in the program remains speculative at present and needs further qualitative investigation. With the pandemic crisis still ongoing, future studies should furthermore distinguish in more detail between different measurement time points to examine how pandemic management and program implementation experiences have improved.

## Conclusion

The results of this analysis indicate that the modified implementation of an 11-month outpatient juvenile weight management program in strict compliance with hygiene rules during the COVID-19 pandemic was as effective as before the pandemic. A combined approach that includes digital content and tailored in-person components could therefore be a future solution to ensure continuity of care in times of social distance.

## Supporting information

S1 File Program plan. (DOCX)

S1 Checklist CONSORT check-list for randomized trials. (DOC)

S1 Table Baseline differences between the program participants during the pandemic. (DOCX)
