# Supplementary material for: Short report: Weight management of children and adolescents with obesity during the COVID-19 pandemic in Germany
Source: PLoS One. 2022 Apr 29;17(4):e0267601. doi: 10.1371/journal.pone.0267601 (PMC9053772; doi:10.1371/journal.pone.0267601)
Supplement: S1 File — (DOCX) [file pone.0267601.s001.docx]

**Program plan “CHILT III”**

# A. General framework

**1. Name of the project**

Children’s Health InterventionaL Trial III (CHILT III) – an outpatient, family-based training program for the treatment of obesity in children and adolescents

**B. Study description**

**1. Scientific objective**

The aim of the CHILT III project is to evaluate the long-term effectiveness of an outpatient family-based program on lifestyle (active/inactive daily and leisure time behavior, nutrition, etc.), anthropometric data/body composition, motor and physical performance (spiroergonometry), selected cardiac parameters (blood pressure, resting ECG) as well as selected biomarkers (lipids, adipokines, cytokines, etc.) and psychosocial factors (quality of life, self-competence perception). For this purpose, the corresponding parameters as well as general personal information and data on the socioeconomic status will be analyzed before and after the project up to a follow-up of two, three and 10 years after the program.

**2. Study Protocol**

**2.1 Sample collective**

A minimum age of 8 years and a maximum age of including 16 years is required for participation. An age-appropriate reading and writing ability is required. The maximum group size is 12 – 15 children.

Inclusion criteria

- Children aged 8-16 years and their parents.

- The participant's BMI exceeds the 97th percentile and/or there are either.

- obesity associated risk factors or
- obesity associated diseases are present

- Sufficient German language skills of the children and parents

- Sufficient motivation of the children and parents to actively participate in the training course

- Normal ability to attend school

- Previous outpatient medical treatment according to the currently available guideline recommendation (e.g. AGA) was unsuccessful.

Exclusion criteria

- Mental disorder or illness (e.g. eating disorder, bulimia)

- Lack of motivation of the children or their parents

- Insufficient group ability

- Obesity that can be traced back to a disease (e.g. Prader-Willi syndrome)

Discontinuation criteria

- No regular participation in the training program

- Participants who have been absent three times in a row without reason and do not show up for the next course unit will be excluded from further participation.
- If course units are cancelled due to illness, an interruption of up to four weeks is accepted.

- Occurrence of mental disorders including eating disorders

- Occurrence of other serious health disorders that make regular participation no longer possible

**2.2 Procedure**

An interdisciplinary team consisting of sports scientists, ecotrophologists, social pedagogues and/or psychologists and doctors is responsible for the organization, planning and implementation of the units.

The program runs for an entire school year. Once a week there is a medical consulting session and a psychosocial/nutritional training session both for children and parents. Twice a week, the children/adolescents participate in a sports program. In addition, there is the possibility of individual meetings.

1. Anamnesis and physical examination (blood pressure measurement, BIA, calipometry)

The physical examinations are presented in more detail below. Body height and weight are taken weekly. All other examinations take place at the beginning of the therapy program, at the end and one year after the intervention program.

At the beginning of the examination, the body height in centimeters is determined. For this purpose, the children/adolescents stand barefoot with their feet in full contact with the ground and in an upright position with their backs against a wall. Contact points are the back of the head, the buttocks and the heels. The head is held straight and the knees are extended. To measure the body height, attention is paid to the exact head position according to the "Frankfurt horizontal". The measurement is taken with a measuring rod fixed to the wall. The body height is determined to the nearest 0.1 cm.

This is followed by the determination of the body weight. Before starting the measurement, a digital scale from Tanita® (Corp., Tokyo, Japan) is tared and the secure standing is checked. Body weight is determined to the nearest 0.1 kg in bare feet and light clothing.

Circumference measurements are taken on the upper arm, thigh as well as abdomen using a non-flexible circumference measuring tape. The measurement takes place directly on the skin. The measured value is recorded to the nearest 0.1 cm.

Skinfold thickness is determined at ten body sites using a caliper (Harpender Skinfold Caliper HSK-BI, British Indicators, West Sussex, England). The measured values are determined to an accuracy of 0.1 mm. Skinfold measurements are taken at the tragus, base of tongue, axilla pectoris, axilla 10th rib, navel, hip, subscapular, triceps, patella, and calf. At each body site, the measurement is repeated three times and then averaged.

The bioimpedance measurement takes place on a couch in a relaxed supine position. The measurement is performed on the extremities of the right side of the body after a three-minute rest. To ensure optimal electrode contact, the backs of the hands and feet must be degreased with a disinfectant spray. The electrodes and measuring clamps are attached at the specified points. When the device is switched on, an imperceptible alternating current is passed through the body and the resistance generated in the body is measured.

Blood pressure is measured under standardized conditions. After a five-minute rest period, the first measurement is taken on the right arm. This is repeated after three and six minutes. To determine the blood pressure, a manual blood pressure monitor with an upper arm cuff from Omron is used, the size of which is adjusted to the upper arm circumference of the test person before the measurement begins.

The resting ECG is also performed in the supine position on a couch. During this procedure, measuring electrodes are applied to the skin at ten points. These points are located on all four extremities and the chest wall. One electrode is placed on each limb, and six electrodes are placed on the torso in a specific order. The electrodes are connected to the ECG machine and the electrical activity of the heart is determined.

2. Questionnaires - lifestyle, quality of life, self-competence, nutrition

Before the beginning and after the end of the intervention, as well as one year after the end, several questionnaires will be used to collect data on active and inactive leisure time behavior, as well as questionnaires on eating behavior (FEV), frequency of consumption (FFL), quality of life (GW-LQ-KJ) and self-esteem (FSK-K). In addition, the questionnaires ask about the social status of the parents, previous illnesses and their physical activity behavior.

3. Laboratory parameters

The two medical examinations are performed by Prof. Dr. med. Dr. Sportwiss. Christine Joisten (formerly Graf). Before the start of the program as well as at the end of the program, a fasting blood sample will be taken to determine the selected blood parameters (glucose, insulin, leptin, proinsulin, adiponectin, resistin, grhelin, visfatin, irisin, fetuina, total cholesterol, HDL, LDL, triglycerides as well as the liver values GOT, GGT and GPT). In addition, the lactate concentration is determined during the spiroergometric examination.

Blood is always taken at the same time of day in the morning between 8 and 9 a.m. after a twelve-hour fasting period following 10 minutes of sitting. The tourniquet is placed approximately one hand width above the puncture site. The tourniquet time is approximately 1 min. After selection of the puncture site, disinfection is carried out with a skin disinfectant approved for this purpose (e.g. Softasept® N). The subsequent puncture is carried out in the direction of the selected vein with a butterfly (at least no. 12) to avoid the risk of hemolysis of the sample material. After successful puncture, the tourniquet is released and blood is collected in the specified order (2 serum tubes/6ml blood, 1 citrate tube/3ml whole blood). After removal of the cannula, the puncture site is closed for a sufficiently long time (approx. 5 minutes) with a swab under sufficient pressure.

All filled blood collection tubes are mixed several times (2 - 3 times) immediately after collection and then stored upright until centrifugation. The serum tubes are centrifuged at 4000 rpm (speed) for 10 min and the serum is pipetted off and frozen at -20°C until further processing.

4. Spiroergometry

During a spiroergometry the child/adolescent sits on a bicycle ergometer, in parallel the respiratory gases are determined by means of a mask over mouth and nose. The subject is supplied with a standardized air mixture and respiratory gases are analyzed simultaneously during inspiration and expiration. In addition to ECG, blood pressure, heart rate and lactate measurement, the power in watts or watts per kg body weight and maximum oxygen uptake (VO2max) or relative to body weight are recorded. The initial load starts at 25 watts and is increased by 25 watts every two minutes until the subjective load limit is reached. The subjective load limit is recorded using the BORG scale. During the stress ECG, the electrodes are applied to the chest wall and extremities (or back) in a specific sequence. Lactate is determined at rest, as well as at the end of each exercise stage. Before the start of lactate sampling, possible contamination on the earlobe is removed with a disinfectant spray. In addition, the earlobe is rubbed by the person to be tested for stimulation. The earlobe is punctured with a blood lancet to obtain a drop of capillary blood, which is collected in an end-to-end glass tube.

5. Reference to the follow-up examinations

The program includes four follow-up examinations, which will take place two (T3), three (T4) and ten (T5) years after completion of the program. During the follow-up examinations, height, weight and circumferences are recorded. At T3, spiroergometry (including blood pressure measurement, resting ECG and bioimpedance measurement) and calipometry will also be performed. In addition, questionnaires on exercise and dietary behavior are given out.

**2.3 Quality assurance and evaluation**

In order to avoid undesirable side effects and to achieve the optimum benefit for participants, quality assurance measures are a matter of course. In addition, they are usually rightly demanded by the health insurance companies involved.

To ensure the quality of the measures, all data collected are entered into an obesity patient follow-up program (APV). This is an IT-based online documentation program in which participant data (age, gender, BMI, BMI-SDS) are collected anonymously and evaluated externally via the University of Ulm. Participation is voluntary, but is required - at least for AGA and KgAS certifications. For more information, see www.a-p-v.de.

In addition, test procedures are presented here or attached, with which the success of the measure can be checked continuously and during therapy (weight status, fitness, well-being), in order - if necessary - to be able to modify the measure.

In 1995, the Institute of Medicine presented the following success criteria for reviewing weight loss programs.

1. The program must fit the participant

In the CHILT III program, we discuss content, prerequisites (e.g. regular attendance, etc.) and individual goals very openly in the initial interview right from the start. Possible obstacles, e.g. long distances or pending changes of school, can lead to premature termination, so organizational barriers should also be addressed and weighed up.

1. The modules of a program must be evidence-based

All necessary disciplines (medicine, nutrition, exercise, psy-chology/education) as well as the integration of the family environment are necessary components of a program; a sufficient duration is required.

1. The program should be successful, as measured by the target variables.

Success is measured by the following parameters (modified according to CHILT III):

- Long-term reduction in weight or BMI and BMI-SDS.
- Improvement of obesity-associated diseases
- consistent adherence to a healthy lifestyle, and
- monitoring of potential adverse effects of the program, e.g. eating disorders.

As a special feature of the CHILT III program, but also included in a contract for integrated care, success is considered to be an increase in motor performance by 10%, e.g. measured as bicycle ergometry or with motor test procedures (verification using the Dordel-Koch Test, DKT, Dordel & Koch 2001).

**3. Possible complications / risks (side effects)**

It is assumed that no additional complications are to be expected. There are only the risks associated with blood sampling. Furthermore, a spiroergometric exercise test is performed. Discontinuation criteria such as subjective exertion, blood pressure increase of >250 mmHg systolic are clearly defined (indications for discontinuation from Lawrenz 2010), so that no complications can be assumed.

**4. Risk-benefit analysis**

The planned studies should provide information on whether targeted lifestyle intervention can have a positive influence on children's weight development, fitness and quality of life. Through corresponding research work, the benefits of an intensive multimodal intervention program are to be examined, from which in turn corresponding recommendations for action for the prevention of overweight and obesity can be derived.

Since this is a crucial issue for obesity therapy, the risk of blood sampling and spiroergometry can be considered low.

**5. Analysis / termination criteria**

Only children and families who explicitly consent to the collection of data will be included in the study.

**6. Patient education, consent forms and anonymization**

Participation is on a voluntary basis. All data will only be collected after obtaining written consent. All patient data are subject to medical confidentiality and are only passed on in anonymous form. During the evaluation, the data are changed in such a way that no reference to the family can be made by third parties. Compliance with data protection regulations is expressly guaranteed.
