# Supplementary material for: Short report: Weight management of children and adolescents with obesity during the COVID-19 pandemic in Germany
Source: PLoS One. 2022 Apr 29;17(4):e0267601. doi: 10.1371/journal.pone.0267601 (PMC9053772; doi:10.1371/journal.pone.0267601)
Supplement: S1 Table — (DOCX) [file pone.0267601.s003.docx]

**S3 Table. Baseline differences between the program participants during the pandemic.**

| Variable | Statistics | Year of participation | | p-value* |
| --- | --- | --- | --- | --- |
|  |  | **2019-20** | **2020-21** |  |
|  |  |  |  |  |
| t1 Age (years) | n  Mean  SD | 18  12.2  2.1 | 10  13.1  2.2 | 0.291 |
| t1 BMI SDS | n  Mean  SD | 18  2.3  0.33 | 10  2.5  0.70 | 0. 325 |
| t1 Relative Physical Fitness (W/kg) | n  Mean  SD | 18  1.8  0.4 | 10  1.7  0.5 | 0.317 |
| t1 Media Consumption (hours/day) | n  Mean  SD | 18  5.3  2.9 | 8  6.2  4.5 | 0.585 |
| t1 HRQOL | n  Mean  SD | 17  75.7  11.9 | 8  86.3  11.4 | 0.055 |
| t1 Social Self-concept | n  Mean  SD | 16  83.9  11.8 | 8  74.9  21.4 | 0.188 |
| Δ BMI SDS | n  Mean  SD | 18  -0.04  0.26 | 10  -0.12  0.35 | 0.521 |
| Δ Relative Physical Fitness (W/kg) | n  Mean  SD | 17  0.0  0.3 | 3  0.2  0.2 | 0.329 |
| Δ Media Consumption (hours/day) | n  Mean  SD | 15  0.5  2.6 | 0 | - |
| Δ HRQOL | n  Mean  SD | 14  -1.6  15.9 | 5  -1.7  15.3 | 0.990 |
| Δ Social Self-concept | n  Mean  SD | 13  -3.8  13.2 | 0 | - |

t1, baseline data; HRQOL, health-related quality of life; SD, standard deviation; Δ, difference in data after 11-month intervention (t2) from baseline data (t1); HRQOL and social self-concept are based on scores ranging from 0 (lowest) to 100 (highest); *Significance values are a result of an independent two-tailed t-test.
